# Disparate mechanisms counteract extraneous CRISPR RNA production in type II-C CRISPR-Cas systems

**DOI:** 10.1093/femsml/uqaf007

**Published:** 2025-05-14

**Authors:** Maximilian Feussner, Angela Migur, Alexander Mitrofanov, Omer S Alkhnbashi, Rolf Backofen, Chase L Beisel, Zasha Weinberg

**Affiliations:** Bioinformatics Group, Department of Computer Science and Interdisciplinary Centre for Bioinformatics, Leipzig University, D-04107 Leipzig, Germany; Helmholtz Institute for RNA-based Infection Research (HIRI), Helmholtz-Centre for Infection Research (HZI), D-97080 Würzburg, Germany; Bioinformatics Group, Department of Computer Science, University of Freiburg, D-79110 Freiburg, Germany; Center for Applied and Translational Genomics (CATG), Mohammed Bin Rashid University of Medicine and Health Sciences (MBRU), Dubai Healthcare City, Dubai P.O. Box 505055, United Arab Emirates; College of Medicine, Mohammed Bin Rashid University of Medicine and Health Sciences (MBRU), Dubai Healthcare City, Dubai P.O. Box 505055, United Arab Emirates; Bioinformatics Group, Department of Computer Science, University of Freiburg, D-79110 Freiburg, Germany; Signalling Research Centres BIOSS and CIBSS, University of Freiburg, D-79104 Freiburg, Germany; Helmholtz Institute for RNA-based Infection Research (HIRI), Helmholtz-Centre for Infection Research (HZI), D-97080 Würzburg, Germany; Medical Faculty, University of Würzburg, D-97070 Würzburg, Germany; Bioinformatics Group, Department of Computer Science and Interdisciplinary Centre for Bioinformatics, Leipzig University, D-04107 Leipzig, Germany

**Keywords:** CRISPR-Cas, extraneous CRISPR RNA, Type II-C CRISPR-Cas systems, CRISPR repeats, RNA secondary structures, mutations

## Abstract

CRISPR-Cas adaptive immune systems in bacteria and archaea enable precise targeting and elimination of invading genetic elements. An inherent feature of these systems is the ‘extraneous’ CRISPR RNA (ecrRNA), which is produced via the extra repeat in a CRISPR array lacking a corresponding spacer. As ecrRNAs would interact with the Cas machinery yet not direct acquired immunity, they pose a potential barrier to defence. Type II-A CRISPR-Cas systems resolve this barrier through the leader sequence upstream of a CRISPR array, which forms a hairpin structure with the extra repeat that inhibits ecrRNA production. However, the fate of ecrRNAs in other CRISPR types and subtypes remains to be explored. Here, we report that II-C systems likely employ disparate strategies to resolve the ecrRNA due to their distinct configuration in comparison to II-A. Applying bioinformatics analyses to over 650 II-C systems followed by experimental validation, we identified three strategies applicable to these systems: formation of an upstream Rho-independent terminator, formation of a hairpin that sequesters the ecrRNA guide, and mutations in the repeat expected to disrupt ecrRNA formation. These findings expand the list of mechanisms in CRISPR-Cas systems that could resolve the ecrRNA to optimize immune response.

## Teaser

The majority of the analyzed type II-C CRISPR-Cas systems have at least one mechanism that functions to inhibit the ‘extraneous’ crRNA and its negative side effects.

## Introduction

CRISPR-Cas systems represent the only known adaptive immune mechanism in bacteria and archaea, enabling these organisms to recognize and eliminate previously encountered plasmids and bacteriophages (Barrangou et al. 2007, Marraffini and Sontheimer, 2008, Seed et al. 2013). This immune memory is encoded within CRISPR arrays, where DNA spacers—derived from fragments of invader genetic material (protospacer)—are stored between conserved repeat sequences. New spacers are added to the CRISPR array between the newest repeat and the leader region, which simultaneously results in the duplication of the newest repeat. Upon reinfection, the CRISPR array is transcribed and processed into individual CRISPR RNAs (crRNAs), each containing a spacer flanked at least on one side by a partial repeat (Brouns et al. 2008, Carte et al. 2008, Deltcheva et al. 2011). These crRNAs are recognized by Cas effector nucleases through the contained repeat. The spacer of the crRNA then guides the nuclease to complementary nucleic acid targets flanked by the protospacer adjacent motif (PAM), leading to their cleavage and, in some cases, inducing extensive RNA or DNA collateral degradation that results in cell dormancy (Koonin et al. 2017). This ensures a defence mechanism upon future encounters with the same invader (Barrangou et al. 2007).

Notably, because the number of repeats in a CRISPR array is always one more than the number of spacers, one repeat—the extra repeat—remains without a spacer counterpart (Liao et al. 2019). In type II systems, this extra repeat is found at the beginning of the transcribed array and, if transcribed and processed, produces an ‘extraneous’ CRISPR RNA (ecrRNA) that lacks a guide sequence corresponding to any prior invader. The production of ecrRNAs poses a potential problem for the CRISPR-Cas immune system, as these nontargeting RNAs could divert Cas proteins away from productive crRNAs, thereby reducing the system’s overall effectiveness. Thus, mechanisms to suppress ecrRNA formation or function may help maintain the efficiency of the CRISPR-Cas defence (Liao et al. 2019, 2022).

**Figure 1.**
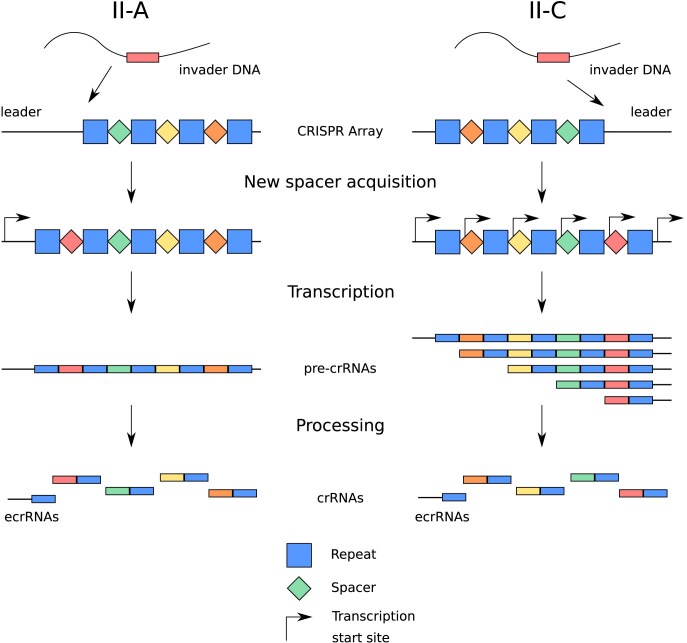
II-A and II-C CRISPR-Cas systems would both derive an extraneous crRNA from the first repeat adjacent to the newest spacer (II-A) or the oldest spacer (II-C). The newest spacer is acquired through the first repeat in the II-A systems (left) or the last repeat in the II-C systems (right). The CRISPR array is transcribed as a single pre-crRNA (II-A) or as multiple pre-crRNAs due to the repeats containing promoters (II-C). The ecrRNA would be formed at the beginning of the array from the first repeat adjacent to the newest spacer (II-A) or the oldest spacer (II-C).

In a preceding analysis, we showed that the II-A CRISPR-Cas leader region suppresses ecrRNA production by forming a hairpin structure with the extra repeat (Liao et al. 2022). In contrast, no such hairpins were identified in type II-C systems. This discrepancy can be attributed to the presence of distinct CRISPR array architectures. In type II-C, spacers are acquired through the last repeat instead of the first repeat, with each repeat possessing its own promoter (Fig. 1) (Dugar et al. 2018, 2013). In type II-C systems, transcription upstream of the CRISPR array could lead to the production of the ecrRNA and weaken the immune response for the same reasons as with II-A. Therefore, we hypothesize that type II-C CRISPR-Cas systems employ different mechanisms to counteract the ecrRNA.

To investigate these regulatory mechanisms, we analysed over 650 type II-C CRISPR-Cas systems. Our analysis revealed three possible strategies to suppress ecrRNA production. First, we found that the extra repeat accumulates significantly more mutations than other repeats in the array, potentially disrupting its interaction with the tracrRNA and hindering ecrRNA processing. Second, we identified Rho-independent terminators (RITs) upstream of type II-C CRISPR arrays that could prevent transcription from extending into the array. Finally, we observed enriched RNA secondary structures such as hairpins formed between the intergenic region upstream of the CRISPR array and the guide region of type II-C ecrRNAs that could interfere with ecrRNA production. The three potential mechanisms expand our knowledge of how the many distinct CRISPR-Cas systems can resolve the ecrRNA and optimize their immune response.

## Methods

### Quality control and division into training and test set

To investigate the fate of the ecrRNA in II-C systems, we used a previously generated data set with more than 650 type II-C systems (Liao et al. 2022). The data set was treated and divided into training and test sets as previously described. In the previous paper, it was assumed that the leader was 180 nt long. This assumption is likely to be wrong because of many potential problems (e.g. undetected mutated repeats or nearby genes). Because of this and other reasons, both sets were subjected to quality control to make sure that the leader and the CRISPR array were not misannotated.

As a first step in quality control, the genomes or contigs of the bacteria in the set of predicted II-C systems were downloaded from the NCBI website via Entrez (Sayers, 2022). Within these genomes, all potential CRISPR-cas cassettes and CRISPR arrays were predicted with CRISPRcasIdentifier (version 1.1.0) and CRISPRidentify (version 1.0) (Mitrofanov et al. 2021, Padilha et al. 2020). Only type II-C CRISPR-Cas loci were used for further analysis. Moreover, the tracrRNA was predicted with CRISPRtracrRNA (version 1.0) (Mitrofanov et al. 2022). With the predicted repeats from the CRISPR array, the leader sequence (for II-A) and upstream intergenic region of the CRISPR array (for II-C) were searched to identify potential missed mutated repeats. For the search, blastn (version: 2.5.0+) (Madden 2002) was utilized with the input parameters -gapopen 2 -gapextend 1 -penalty -1 -reward 1 -word_size 5. A potential hit from blastn was considered a mutated repeat if it was found 20–45 nt away from the last repeat, had a sequence similarity of above 70% to the consensus repeat, and had an e-value below 0.5. For each of these potential repeats, an empirical e-value was calculated by searching for hits with a better blast score 1000 times in a dinucleotide shuffled leader sequence or upstream intergenic region. A potential repeat was classified as a mutated repeat if its empirical e-value was below 0.01. While this threshold may not eliminate all false positives, a more stringent threshold would significantly reduce sensitivity. The mutated repeat was added to the CRISPR arra, y and the leader sequence or upstream intergenic region was updated. As a last step in the quality control, the 180 nt leader was shortened if the upstream gene was closer than 180 nt to the CRISPR array. Leader sequences shorter than 30 nt were discarded.

### Statistical testing for RNA secondary structures in the leader sequence

The statistical tests were conducted in accordance with the methodology outlined in the preceding paper (Liao et al. 2022). In contrast to the previous approach, which focused on the potential helices between the leader and the extra repeat, this study examined the potential helices within the upstream region of the CRISPR array and between the upstream region of the CRISPR array and the spacer of the ecrRNA (assumed to be 25 nt upstream of the extra repeat). In the training data set (*N* = 147 II-C systems), the helix parameter of 12 base pairs with up to 3 unpaired nucleotides in a bulge or internal loop for the helix within the leader and 8 base pairs with up to 2 unpaired nucleotides in a bulge or internal loop for the helix between the leader and the ecrRNA spacer were identified as the most promising parameters. Because of that, these parameters were used to analyse the test data set (*N* = 113 II-C systems). As a negative control, type II-A systems were used and also split into training (*N* = 35 II-A systems) and test data sets (*N* = 28 II-A systems).

### Analysis of the mutation pattern in II-C CRISPR arrays

In order to analyse the mutation patterns, all type II-C and II-A CRISPR arrays which passed the quality control were utilized. Prior to the analysis, redundant CRISPR arrays were filtered out using CD-HIT (Version 4.8.1) (Fu et al. 2012) with a sequence identity threshold of 1 and the repeat sequences of all repeats in the array concatenated to a single string as input. The initial stage of the analysis entailed the removal of all repeats that exhibited a discrepancy in length of either two or more nucleotides in comparison to the consensus repeat sequence observed in each CRISPR array. Subsequently, the repeat sequences of each CRISPR array were aligned with the multiple sequence alignment tool Muscle (version 3.8.1551). Thereafter, each aligned repeat sequence was pairwise aligned to the consensus repeat (taken from the CRISPRcasIdentifier predictions) with the pairwise alignment tool Needle (input parameter: -gapopen 10 -gapextend 4 -endweight -endextend 4) (Madeira et al. 2024). In each pairwise alignment, gaps and mismatches were counted as mutations.

### RIT prediction

The RITs in the leader regions were predicted with RNIE (version 0.01) (Gardner et al. 2011) using the default parameters and the leader sequence as the input file. In order to identify RITs, the covariance models ‘super43-45-seed’, ‘erpin-rho’, ‘fast’, ‘seed-b-14’, and ‘genome’ from RNIE were employed.

To ascertain whether RITs are enriched in the leader region of type II-C CRISPR arrays, we calculated the probability of an RIT being present in an intergenic region exceeding 30 nt for each bacterium in our training and test data set. To this end, all potential proteins in the downloaded genome were annotated with Prodigal (version 2.6.3) (Hyatt et al. 2010) and all intergenic regions exceeding 30 nt were extracted from the annotation file. As a second step, RNIE was employed to predict all RITs in the genome, utilizing the default parameters with the exception of the ‘–genome’ flag and the genome file as the input. Subsequently, the number of RITs in the previously extracted intergenic regions was counted and divided by the total number of intergenic regions. The resulting estimate of the expected number of RIT was then aggregated across all analysed genomes and divided by the total number of genomes to derive the average number of expected RITs in intergenic regions. The same procedure was employed for the combined test and training data set from type II-A, which served as a negative control. Furthermore, the number of RITs in the upstream intergenic region of the CRISPR array was counted in order to facilitate a comparison with the overall expected number of RITs.

### Calculation of the interaction energy between CRISPR repeats and the tracrRNA

In order to test the hypothesis that the interaction between tracrRNA and the extra repeat is impaired by mutations identified in the extra repeat, a comparison was made between the interaction energy of the consensus repeat and the extra repeat with the predicted tracrRNA. The same data set used for the mutation pattern analysis was employed for this analysis, except that redundant systems where the tracrRNAs exhibited a sequence similarity above 70% (according to CD-HIT) were removed. This was done to eliminate high correlations among the samples, which is a prerequisite for conducting a Mann–Whitney U test to ascertain any differences between the consensus and extra repeats. Nevertheless, our methodology is predicated on the assumption that the sequences under examination are evolutionarily related. It was anticipated that the removal of sequences with a similarity of over 70% would address this issue. It was not possible to pursue a more rigorous elimination of analogous sequences (for instance, at 60% identity) due to the relatively limited number of II-C systems currently accessible. The interaction energy between the remaining repeats and the relevant tracrRNA was calculated using IntaRNA (version 2.3.1) (Raden et al. 2018) with the default parameters. The Mann–Whitney U test was employed to ascertain whether there were statistically significant differences in the interaction energy between the consensus and extra repeats.

### Strains, plasmids, and growth conditions

*Escherichia coli* cells were grown at 37°C in Luria–Bertani (LB) broth (5 g/l NaCl, 5 g/l yeast extract, and 10 g/l tryptone) with shaking at 220 rpm or on LB agar plates (LB broth, 18 g/l agar). *Escherichia coli* strain TOP10 was used for plasmid cloning. Antibiotics were added where appropriate at final concentrations of 100 μg/ml for ampicillin, 34 μg/ml for chloramphenicol, and 50 μg/ml for kanamycin.

All primers used in this work were ordered from Integrated DNA Technologies. Q5 site-directed mutagenesis kit (New England Biolabs) was used for small insertion and nucleotide substitution.

For the flow cytometry and quantitative Reverse Transcription PCR (RT-qPCR) analysis, three GFP-expressing plasmids (AM377-9) and one no-GFP-control plasmid (AM380) were created. Briefly, to test the RIT upstream of the Type II-C CRISPR array, 84 nucleotides upstream of the first repeat together with 5 nucleotides of the first repeat from the type II-C system of *Campylobacter jejuni* NCTC 11168 were cloned between a promoter and an RBS of the *gfp* gene (AM377). In addition, the plasmids were created with the mutations disrupting the stem-loop (AM378) or uracil-stretch (AM379) of the transcript.

To test the activity of the predicted ecrRNA from the type II-C system of *C. jejuni* NCTC 11168, a plasmid expressing an ecrRNA in the form of the single-guide RNA, or esgRNA, was created (AM003) for the TXTL assay. Additionally, the nontargeting control plasmid (NT sgRNA, CB341) was created as well as the plasmids with the mutation in the esgRNA disrupting the stem-loop structure in the spacer (mesgRNA, AM006) or with the mutation of the first nucleotide of the repeat from A to G in the esgRNA [esgRNA(A→G), AM004]. The GFP variant deGFP was used as the fluorescent reporter and was encoded on a plasmid with the ecrRNA target (AM390). Plasmid with CjCas9 (CBS117) was used as a nuclease plasmid.

### Flow cytometry analysis

Overnight cultures of *E. coli* CB414 cells harbouring the GFP-expressing plasmids or no-GFP-control were back diluted 50 times in LB medium supplemented with kanamycin, and shaken at 220 rpm at 37°C to ABS600 = ∼0.8. Next, cultures were diluted 1:20 in 1x phosphate-buffered saline (PBS) and analysed on an Accuri C6 Plus flow cytometer with BD CSampler Plus (Becton Dickinson), a 488-nm laser and a 530/30-nm bandpass filter. Forward scatter (cutoff, 11 500) and side scatter (cutoff, 600) were used to eliminate noncellular events. The mean fluorescein isothiocyanate-A value of 30 000 events within a gate set for live *E. coli* cells was used for data analysis after subtraction of cell autofluorescence. The assay was performed in three biological replicates.

### RNA extraction, RT-qPCR

Overnight cultures of *E. coli* CB414 cells harbouring the GFP-expressing plasmids were back diluted 100 times in LB medium supplemented with kanamycin, and shaken at 220 rpm at 37°C to ABS600 = ∼0.8. 5 ml of culture was mixed with 1 ml of stop solution (95% EtOH + 5% phenol), and harvested with centrifugation at 5000 × *g* for 10 min at 4°C.

For total RNA extraction, the cell pellet was resuspended in 600 µl 0.5 mg/ml lysozyme in TE pH 8.0; 60 µl 10% w/v SDS was added; the mix was incubated at 64°C for 1 min. Next, 66 µl 3 M NaOAc, pH 5.2 and 750 µl phenol were added to the cell lysate, the mix was incubated at 64°C for 6 min. Then, the samples were centrifuged at 15 000 × *g* for 15 min at 4°C. The aqueous layer was transferred into new tubes and mixed with 750 µl of chloroform. After another centrifugation at 15 000 × *g* for 15 min at 4°C, the aqueous layer was mixed with 1.4 ml (30:1) EtOH: NaAcetate (from stock 3 M pH 6.5) to precipitate total RNA at −20°C overnight. After precipitation, the samples were centrifuged at 15 000 × *g* for 30 min at 4°C. The supernatant was removed, and the RNA pellet was washed with 70% EtOH and air-dried. The RNA pellet was resuspended in water and purified with RNA Clean and Concentrator columns-25 (Zymo Research), including in-column DNase I treatment step.

50 ng of the DNase I-treated total RNA was used for the RT-qPCR with the primers to *gfp* and *gapA* genes. *gapA* mRNA levels were used to normalize *gfp* mRNA levels across the strains harbouring different *gfp* constructs. cDNA synthesis and Reverse Transcription PCR (RT-PCR) was performed using iTaq™ Universal SYBR® Green One-Step Kit (Bio-Rad) and measured with CFX Opus 96 Real-Time PCR System (Bio-Rad). The assay was performed in three biological replicates.

### Cell-free transcription–translation reactions

Cas9, sgRNA, and deGFP-encoding reporter plasmids were added into myTXTL Sigma 70 Master Mix (Arbor Biosciences) at a final concentration of 3 nM, 0.5 nM and 0.5 nM, respectively. All components were transferred into a 96-well plate. The DNA cleavage assay was performed in 5-μl reactions by measuring fluorescence on a Synergy Neo2 plate reader (BioTek) using a 96-well V-bottom plate (Corning Costar) with an excitation filter of 485 nm and an emission filter of 528 nm. Time courses were run for 16 h at 29°C with an interval of 3 min between reads. The assay was performed in two independent replicates.

## Results

### Mutations in extra repeats in type II-C could interfere with the interaction between the ecrRNA and the tracrRNA

The oldest repeat in a CRISPR array tends to accumulate more mutations over time than other repeats (Liao et al. 2019, Horvath et al. 2008, Fehrenbach et al. 2024). Our primary objective was to determine whether mutations in the extra repeat are common in type II-C systems and whether these mutations could impact the interaction between tracrRNA and pre-ecrRNA, potentially affecting ecrRNA processing. In type II-C CRISPR-Cas systems, new spacers are acquired downstream of the CRISPR array compared to type II-A systems (Hooton and Connerton, 2015, He et al. 2018). This structural distinction allows the extra repeat in type II-C arrays to be mutated without disrupting spacer acquisition. Building on this knowledge, we began our analysis by investigating the mutation frequencies of the extra repeat.

**Figure 2.**
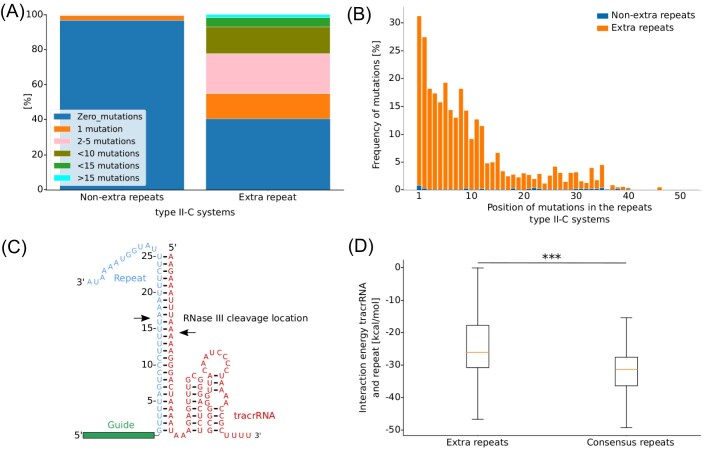
Mutation in the extra repeat weakens the interaction energy between extra repeat and tracrRNA. (A) Comparison of the numbers of mutations present in nonextra (left) and extra (right) repeats in CRISPR arrays from type II-C. 364 nonredundant type II-C CRISPR arrays were analysed. Absolute numbers of repeats per category are shown in Table S1. (B) Position of the mutations in the extra and nonextra repeat. 364 type II-C CRISPR arrays were analyzed with a total of 7208 repeats. (C) Interaction between crRNA and tracrRNA of *C. jejuni* NCTC 11168. RNase III cleavage location is marked with black arrows. (D) Interaction energy between tracrRNA and the extra (left) and the consensus repeat (right). 69 type II-C CRISPR-Cas systems were analyzed (*** *P* < 1e^−5^).

After removing redundant systems—those with identical repeat sequences—we focused on a dataset of 357 unique type II-C systems. We then compared the mutation frequency between the first repeat (which is both the oldest repeat and the extra repeat) and the other repeats within each CRISPR array. Our analysis revealed that 60% of the extra repeats contained at least one mutation, whereas only 5% of the nonextra repeats exhibited mutations (Fig. 2A). This finding indicates that the extra repeat in type II-C CRISPR-Cas systems accumulates more mutations than other repeats in the array.

Following this observation, we explored whether these mutations are likely to interfere with the forming or cleavage activity of the ecrRNA: tracrRNA: Cas9 complex. For that, we analyzed the positioning of the mutations in the extra repeat. Interestingly, most mutations occurred within the first 15 nts, which is the region interacting with Cas9 after the processing of RNase III (Fig. 2B and C). Previously, it was shown that mutations in this region inhibit DNA cleavage if base-pairing between tracrRNA and repeat is disrupted (Jiao et al. 2021, Briner et al. 2014). Accordingly, we found that the interaction length and the interaction energy between the tracrRNA and the extra repeat were shorter and weaker, respectively than between the tracrRNA and the consensus repeat (Fig. 2D and Fig. S1), suggesting that these mutations may interfere with tracrRNA:ecrRNA interactions and, potentially, ecrRNA function.

Building on the *in silico* predictions, we examined whether the observed differences in interaction energy between extra repeats (median: −26 kcal/mol) and consensus repeats (median: −31 kcal/mol) were statistically significant. Using the Mann–Whitney U test, we found a significant difference in the distributions of the interaction energy between extra repeats and consensus repeats (*P*-value: 5.1 × 10^−6^). Although mutations in the extra repeat that weaken interaction with the tracrRNA cannot definitively be attributed to positive selection (Fig. S3), these mutations tend to cluster in a region critical for DNA targeting. These findings suggest the presence of an additional regulatory mechanism in type II-C systems that may inhibit the ecrRNA through altered repeat: tracrRNA interactions and disrupted Cas9 cleavage activity.

While over 60% of extra repeats exhibit mutations, this proportion is lower than the mutation rate observed in the oldest repeat of type II-A systems (∼75%) (Fig. S2). This observation raises the possibility that type II-C extra repeats might experience a reduced mutation rate and that mutations in the extra repeat are not the dominant strategy to inhibit the ecrRNA. Consequently, we sought to explore alternative mechanisms that might regulate ecrRNA formation.

### RITs insulate the CRISPR array from outside transcription

**Figure 3.**
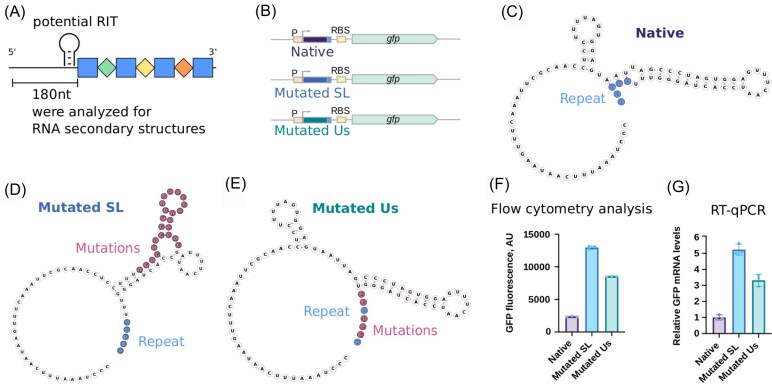
RNA secondary structures found in the intergenic region upstream of the II-C CRISPR arrays can terminate transcription. (A) Up to 180 nt upstream of over 650 the type II-C CRISPR arrays were analyzed for RNA secondary structures and potential RITs were found. The potential RIT in *C. jejuni* NCTC 11168 directly upstream of the extra repeat was further characterized. (B) The GFP reporter construct design. 84 nucleotides upstream of the first repeat together with 5 nucleotides of the first repeat from *C. jejuni* NCTC 11168 were cloned into the GFP-expressing plasmid between the promoter and the RBS of the *gfp* gene. Besides the native sequence, the sequences with the mutations disrupting the stem-loop (SL) or uracil-stretch (Us) of the transcript were tested. (C) Putative structure of the native transcript, including five repeat nucleotides, from *C. jejuni* cloned into the GFP reporter plasmid. (D) Putative structure of the transcript as in (B) with the mutations disrupting the stem-loop. (E) Putative structure of the transcript as in (B) with the mutations disrupting the uracil-stretch. (F) The GFP fluorescence was measured in *E. coli* cells transformed with the GFP reporter plasmids using a flow cytometer. The mean and standard errors of three independent measurements are plotted. Termination of transcription leads to lower fluorescence. (G) The GFP mRNA levels were measured in *E. coli* cells transformed with the GFP reporter plasmids using RT-qPCR. The mean and standard errors of three independent measurements are plotted.

Prior work on the ecrRNA of type II-C systems did not reveal a hairpin formed between the intergenic region upstream of the CRISPR array and extra repeat (Liao et al. 2022). However, RNA secondary structures could exist further upstream that could also impact ecrRNA transcription or formation. We, therefore, adapted our prior RNA secondary structure search method (Liao et al. 2022) to analyze up to 180 nts upstream of over 650 type II-C CRISPR arrays (Fig. 3A). In these upstream regions, we observed a 12-bp helix with up to three unpaired nucleotides in a bulge or internal loop significantly more often than in a randomized control (*P* = 8 × 10^–5^). Furthermore, RNA secondary structures were not significant in the upstream region of II-A CRISPR arrays (*P* = .41), indicating the potential importance of the upstream RNA secondary structure for type II-C. A subset of the RNA secondary structures upstream of II-C arrays were identified as RITs, which are hairpins with a poly-uracil tail that prevent RNA polymerase from further transcription (Gardner et al. 2011). These RITs could insulate the CRISPR array from transcription from outside the CRISPR array, e.g. from upstream genes.

To test whether the putative RITs can disrupt transcription, we cloned a predicted RIT directly upstream of the type II-C CRISPR array from *C. jejuni* NTCT 11168 into a GFP reporter system (Fig. 3). In addition, we created two constructs with mutations either disrupting the predicted stem-loop or in the uracil stretch following the stem-loop. Both mutations elevated GFP expression in *E. coli* cells, confirming transcriptional repression by the native structure.

As a subsequent step, we sought to ascertain whether upstream RITs were a distinctive attribute exclusive to type II-C systems. Again, compared to II-A arrays, RITs occur more often in the intergenic region upstream of II-C CRISPR arrays (12%, or 31 observed RITs out of 260 systems) than in type II-A arrays (3%, or 2 observed RITs out of 63 systems) (Table 1). This prompts the following question: do RITs not only occur more frequently upstream of type II-C CRISPR arrays compared to type II-A CRISPR arrays, but also more frequently than expected by chance? In order to answer this question, we compared the number of RITs observed in non-CRISPR array-related intergenic regions with the number of RITs observed in the intergenic region upstream of the type II-C CRISPR array for each bacteria with an analyzed type II-C system. The results of this analysis revealed the presence of RITs in 11% of the intergenic regions upstream of type II-C CRISPR arrays, in contrast to 12% observed in non-CRISPR array-related intergenic regions. This indicates no statistically significant enrichment of RITs upstream of type II-C arrays. However, the presence of RITs in some type II-C systems suggests a potential role in preventing external transcription from entering the CRISPR array.

**Table 1.** Analysis of the intergenic regions and their RIT content upstream of the CRISPR array in type II-A and II-C.

	Average length intergenic region	Observed RIT	Systems analyzed	Ratio	Expected ratio
II-A	68 nt	2	63	0.03	0.21
II-C	247 nt	31	260	0.11	0.12

RITs were predicted with RNIE (Gardner et al. 2011). Ratio equals observed RITs divided by analyzed systems. The expected ratio indicates the number of found RITs in intergenic regions (>30 nt) divided by the number of these intergenic regions found in the bacteria in the training and test data set.

### Helices in the extraneous crRNA guide could interfere with the processing or the target recognition of the extraneous crRNA

**Figure 4.**
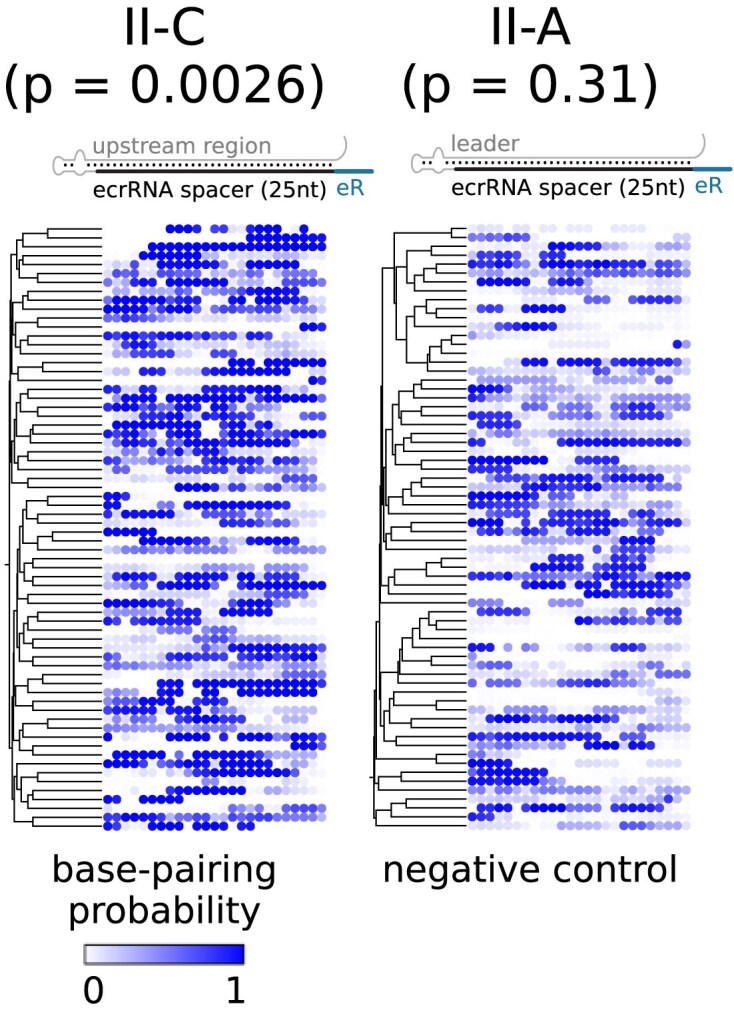
Predicted helix formation between the 25 nt ecrRNA spacer and the upstream region. Each dot represents one nucleotide of the ecrRNA and its base-pairing probability with the leader. Left: type II-C with an aggregated *P*-value of .0026, right: type II-A with an aggregated *P*-value of .31 as the negative control (eR: extra repeat, ecrRNA: extraneous CRISPR RNA).

It is noteworthy that, even after the removal of systems with predicted RITs, a significant *P*-value of .001 was observed for the presence of hairpin structures in the upstream region of type II-C CRISPR arrays. This observation suggests that these RNA secondary structures may possess alternative functions e.g. impairing crRNA-guided targeting as a hairpin in the spacer (Creutzburg et al. 2020, Riesenberg et al. 2022). To test if a similar mechanism is present in type II-C CRISPR-Cas systems to prevent ecrRNA targeting, we modified our previous statistical test (Liao et al. 2022) to include only RNA secondary structures with base pairing between the 25 nt spacers of ecrRNA and the upstream region. With the help of the modified test, we found that 8-bp helices with up to two unpaired nucleotides in a bulge or internal loop occur significantly more often than expected by chance (*P* = .0026, after removing all systems with predicted RITs). In contrast, similar hairpins in type II-A systems are not significantly enriched (*P* = .31; Fig. 4).

**Figure 5.**
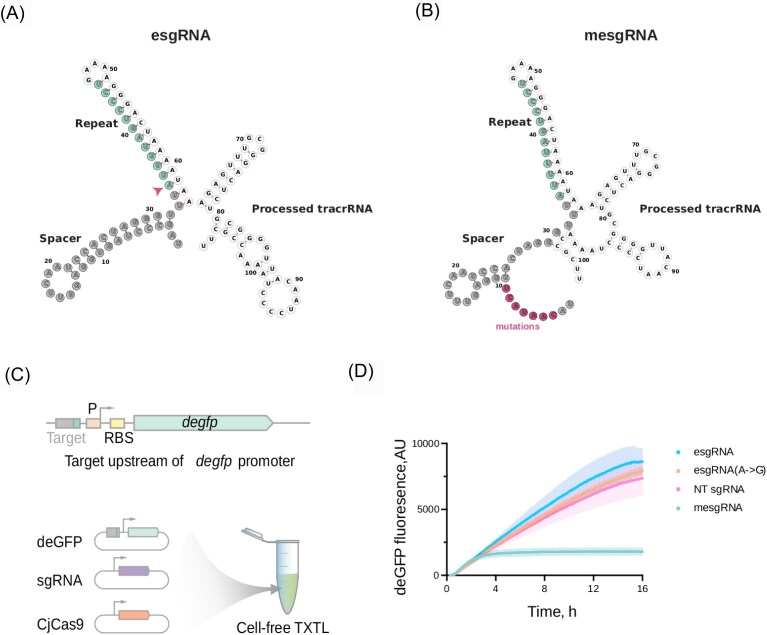
Stem-loop in the ecrRNA spacer of *C. jejuni* NCTC11168 inhibits targeting of ecrRNA. (A) Putative structure of the extraneous single-guide RNA (esgRNA) from *C. jujeni* NCTC 11168 predicted with RNAfold. This structure shows the predicted inhibitory hairpin in the spacer, as well as the typical repeat and tracrRNA structures for type II-C. The first nucleotide of the repeat is an A (marked with an arrow) whereas all other repeats of the array start with a G. (B) Putative structure of the extraneous sgRNA with mutations disrupting the stem-loop structure in the spacer (mesgRNA) from *C. jujeni* NCTC 11168 predicted with RNAfold. (C) Applying the TXTL assay to characterize the targeting activity of the extraneous sgRNA and the impact of the mutations on it. (D) The targeting activity assay was performed in TXTL to compare esgRNA, esgRNA(A→G) (with the repeat starting with a G instead of an A), and mesgRNA. NT sgRNA served as a nontargeting control. The mean (dark-coloured lines) and standard errors (lighter-coloured bars) of two independent measurements are plotted. When the target is recognized on a deGFP-expressing plasmid, the nuclease cleaves the plasmid, which leads to lower fluorescence.

We hypothesize that these helices could interfere with the processing or targeting of the extraneous crRNA. To test the targeting activity of the predicted ecrRNA in the type II-C array from *C. jejuni* NTCT 11168, we created a plasmid expressing the ecrRNA in a fused form to tracrRNA, which we called the extraneous sgRNA (esgRNA). The targeting potential of the esgRNA was tested in a cell-free transcription–translation (TXTL) system using DNA constructs encoding the *C. jejuni* Cas9 (CjCas9), an RNA guide, and a GFP reporter harbouring a target sequence flanked by a recognized PAM (Jiao et al. 2021) (Fig. 5). GFP fluorescence is then measured over time as a readout of DNA targeting by CjeCas9. A mutated version of the esgRNA (mesgRNA) was included that disrupts the hairpin while maintaining the targeting portion of the guide region. Additionally, a variant was tested where the first nucleotide of the esgRNA was modified from A to G, consistent with the consensus repeat sequence.

We found the esgRNA did not reduce GFP fluorescence compared to a nontargeting sgRNA, whereas the mutated esgRNA significantly reduced GFP fluorescence by 4-fold (*P* = .027). The reduced GFP silencing for the esgRNA versus the mesgRNA is in line with reduced targeting efficiency due to the structure of the spacer, whether through reduced binding to Cas9 or reduced DNA target recognition. We also noticed that the four-spacer array of *C. jejuni* NCTC11168 consists of four identical repeats and one with a mutation. The mutated repeat is the first repeat (the oldest one in the array) and it starts with A when the other ones start with G. We decided to test whether this mutation could be another factor facilitating inhibition of ecrRNA activity. We mutated the first nucleotide of the repeat in esgRNA from A to G, however, this mutation did not restore the activity of the esgRNA. These results confirm that the identified hairpin inhibits Cas9-mediated DNA targeting through the ecrRNA.

### Most type II-C systems possess at least one mechanism that can inhibit the ecrRNA

**Figure 6.**
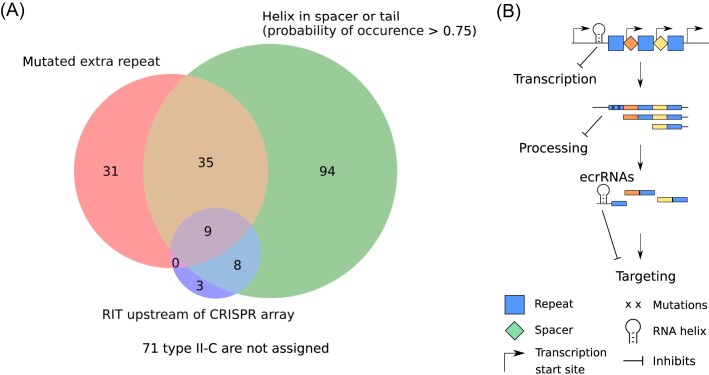
Most of the type II-C systems can be assigned to one of the mechanisms potentially preventing the ecrRNA. (A) Systems with mutated repeat are represented by the left circle, with helices in the upstream intergenic region overlapping the ecrRNA spacer or not by the right circle, and with RITs by the lower circle. The numbers account for II-C systems assigned to each subset. 71 out of 260 systems are not assigned to the potential mechanisms. (B) Schematic overview of the different mechanisms and how they prevent the ecrRNA.

After finding three mechanisms that can interfere with the ecrRNA, we were interested in how many type II-C systems possess at least one of the mechanisms. We, therefore, assigned each system from the training and test data set to the mechanism that is present in that system. We chose three different thresholds to determine whether one of the mechanisms could be present in each type II-C system. For the extra repeat mutations, we chose the threshold of at least two mutations in the extra repeat. For the RITs, we chose the prediction from RNIE. Finally, for the helix in the intergenic region upstream of the CRISPR array, either overlapping the ecrRNA spacer or not, we chose a probability of occurrence above 75%. With these thresholds, we found that 73% of type II-C systems have a potential mechanism present to inhibit the ecrRNA. Furthermore, the largest overlap between two different mechanisms can be found between the RIT and RNA secondary structures groups (17 out of 20 type II-C systems with RIT). This can be explained by the fact that an RIT is counted as a helix in the ecrRNA spacer if the RIT occurs within 25 nt of the extra repeat. Moreover, two-thirds of type II-C systems that have a helix in their ecrRNA guide or the intergenic region upstream of the CRISPR array do not have a mutated extra repeat or an RIT, and only 3.5% of all type II-C systems possess all three (Fig. 6).These findings suggest that the proposed mechanisms may cooccur and potentially synergize with one another. In conclusion, our bioinformatics analysis, supported by experimental evidence, provides new insights into multiple mechanisms that could inhibit ecrRNA activity in type II-C CRISPR-Cas systems, offering a deeper understanding of their regulatory processes.

## Discussion

In the ongoing evolutionary arms race between bacteria and their phages, bacteria have developed defence mechanisms like CRISPR-Cas systems to enhance their survival (Hampton et al. 2020). The CRISPR-Cas system relies on a complex interplay of mechanisms, including spacer acquisition and spacer prioritization, which can be at odds. On the one hand, bacteria must acquire new spacers to combat novel bacteriophages, while on the other, an overabundance of spacers in the CRISPR array can weaken the immune efficacy of individual spacers (McGinn and Marraffini, 2016). To maintain optimal functionality, these systems may benefit from mechanisms that deprioritize nontargeting crRNAs, such as ecrRNAs (Liao et al. 2019, 2022). In this study, we identified three potential mechanisms by which type II-C CRISPR-Cas systems might inhibit the production or function of ecrRNAs.

First, we observed that the extra repeat, which is also the oldest repeat within type II-C CRISPR arrays, harbours significantly more mutations compared to other repeats in the array. The degradation of the oldest repeat is a commonly observed fact for CRISPR arrays (Seed et al. 2013, Liao et al. 2019, Horvath et al. 2008, Fehrenbach et al. 2024). In the case of type II-C, mutations were observed in the repeat region critical for DNA targeting by Cas9 and our mathematical modelling indicates that these mutations could impair the interaction between the tracrRNA and the extra repeat, thereby impairing the processing and subsequent utilization of ecrRNAs in type II-C. This suggests a mechanism of self-regulation within the CRISPR-Cas system that prevents the accumulation of nonfunctional crRNAs. Interestingly, comparative analysis may suggest that type II-A CRISPR arrays exhibit a higher mutation frequency in their oldest repeats compared to the extra repeats in type II-C systems. Thus, there might be other reasons for mutations in these extra repeats unrelated to the suppression of the ecrRNA. However, these discrepancies with our hypotheses could be explained by the fact that the extra repeat needs to retain its function as a promoter to transcribe the array’s first spacer (Dugar et al. 2013). Moreover, type II-C systems may rely on additional mechanisms to suppress ecrRNA activity, which could be critical for maintaining the system’s overall defence efficiency.

We further demonstrated that type II-C CRISPR arrays contain a considerably higher number of RITs in their upstream intergenic regions compared to type II-A arrays. Our experimental characterization of these RITs showed that they can inhibit downstream transcription, thereby potentially suppressing the transcription of the ecrRNA and the overproduction of older crRNAs. This, in turn, may enhance the immune response in bacteria containing type II-C CRISPR systems. However, our attempts to demonstrate a statistically significant enrichment of RITs upstream of type II-C arrays compared to other intergenic regions across bacterial genomes yielded inconclusive results. One possible explanation for the inconclusive frequency comparison of RITs is the inherent difficulty in detecting these elements, particularly in less well-characterized bacterial species. Given that RITs may vary in sequence and structure, current bioinformatic tools might overlook some of these elements (Gardner et al. 2011, Di Salvo et al. 2019). Further research employing more refined detection methods could help clarify the prevalence and functional significance of RITs in type II-C CRISPR systems.

In addition to these mechanisms, we identified a statistically enriched RNA secondary structure between the spacer of type II-C ecrRNAs and their upstream leader regions. Our experimental data suggest that these hairpin structures can inhibit Cas9 binding and/or the targeting potential of ecrRNAs. Similar negative effects on targeting efficiency have been reported for other type II systems in which RNA secondary structures form within or adjacent to the spacer (Creutzburg et al. 2020, Riesenberg et al. 2022). Notably, the hairpin we analysed experimentally is predicted to function as an RIT, making it challenging to distinguish whether the reduced targeting efficiency is due to the hairpin itself or the RIT.

Most type II-C ecrRNAs could be inhibited by one or more of the described mechanisms, yet the mechanisms could combine to increase the inhibition of the ecrRNA. For instance, the strain *Haemophilus parainfluenzae T3T1* has all three mechanisms present. Nevertheless, approximately a quarter remain unexplained by these pathways. This discrepancy could be attributed to variations in CRISPR-Cas system configurations. For instance, some systems may not require ecrRNA inhibition due to significantly higher relative expression levels of targeting crRNAs or the lack of upstream transcription if the upstream gene is distant or encoded on the opposite strand. However, other mechanisms may await discovery.

Overall, our findings suggest that one or the combination of all three regulatory mechanisms—mutational degradation of the extra repeat, RIT-mediated transcriptional interference, and RNA secondary structure formation—may be sufficient to prevent the formation, processing, and targeting of ecrRNAs in type II-C CRISPR-Cas systems. This study builds on previous research and extends our understanding of ecrRNA inhibition by identifying multiple potential layers of regulatory control within type II-C systems. By mitigating the production of nontargeting crRNAs, these regulatory strategies could play a crucial role in maintaining the system's defensive efficiency. Our analysis provides a basis for detailed studies to determine the precise biological importance of each mechanism in specific systems. Furthermore, these insights into CRISPR array regulation could inform future biotechnological applications, particularly in the development of tools that leverage type II-C CRISPR-Cas systems for precise genetic editing and regulation.

## Supplementary Material

uqaf007_Supplemental_Files

## Data Availability

The strains, plasmids, and oligonucleotides underlying this article are available in the article and in its online supplementary material (Supplementary Data 2). Moreover, the analyzed type II-A and II-C systems are available on GitHub (https://github.com/MaxFeussner/ecrRNA_type_II-C).
