# Supplementary material for: Disparate mechanisms counteract extraneous CRISPR RNA production in type II-C CRISPR-Cas systems
Source: Microlife. 2025 May 14;6:uqaf007. doi: 10.1093/femsml/uqaf007 (PMC12080349; doi:10.1093/femsml/uqaf007)
Supplement: uqaf007_Supplemental_Files [file uqaf007_supplemental_files.zip › Supplementary-Data-1--supplementary-figures-and-tables.pdf]

Supplementary Data 1: supplementary figures and tables

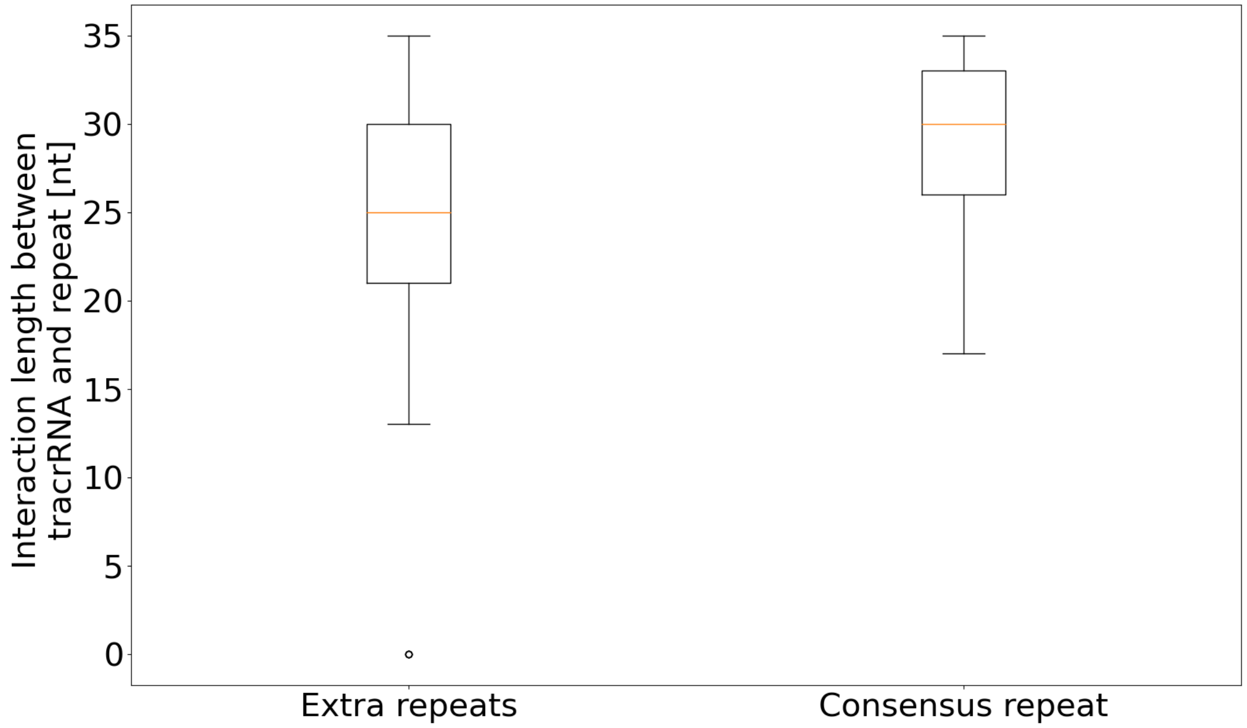

Figure S1: Interaction length between tracrRNA and extra/consensus repeat. 69 type II-C CRISPR-Cas systems were analyzed.

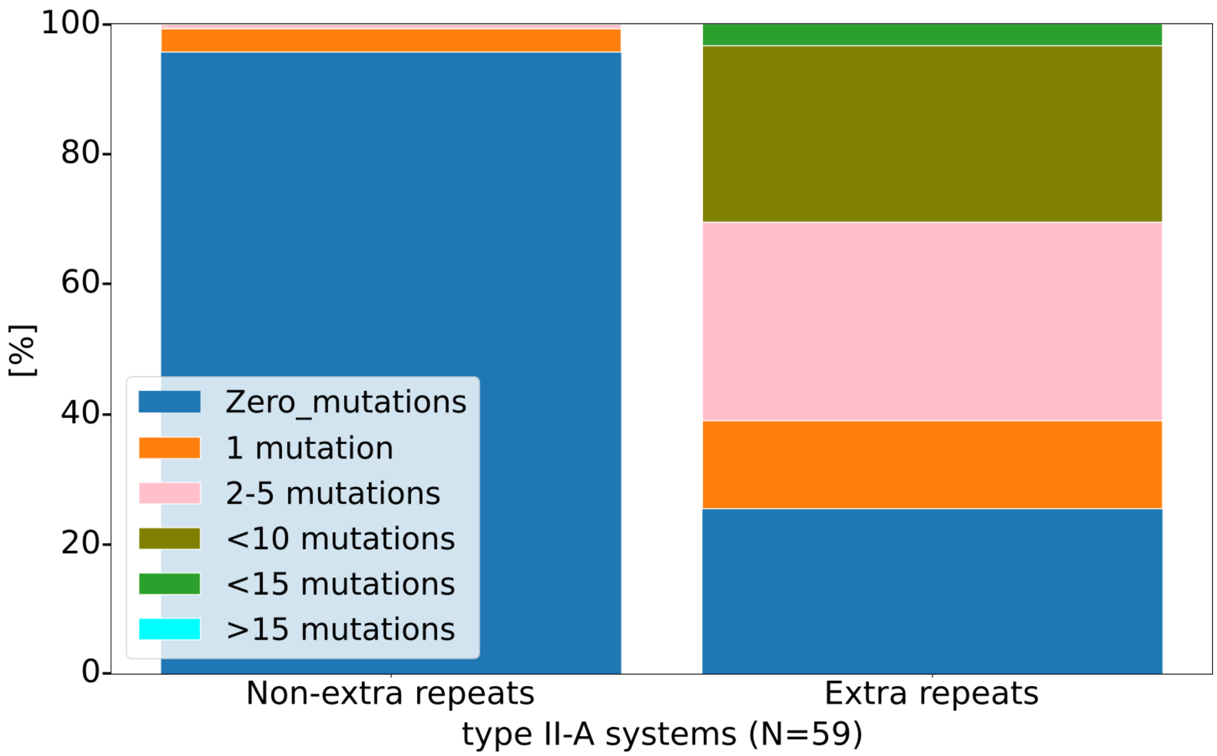

Figure S2: Comparison of the numbers of mutations present in non-extra (left) and extra (right) repeats in CRISPR arrays from type II-A. 59 non-redundant type II-A CRISPR arrays were analysed.

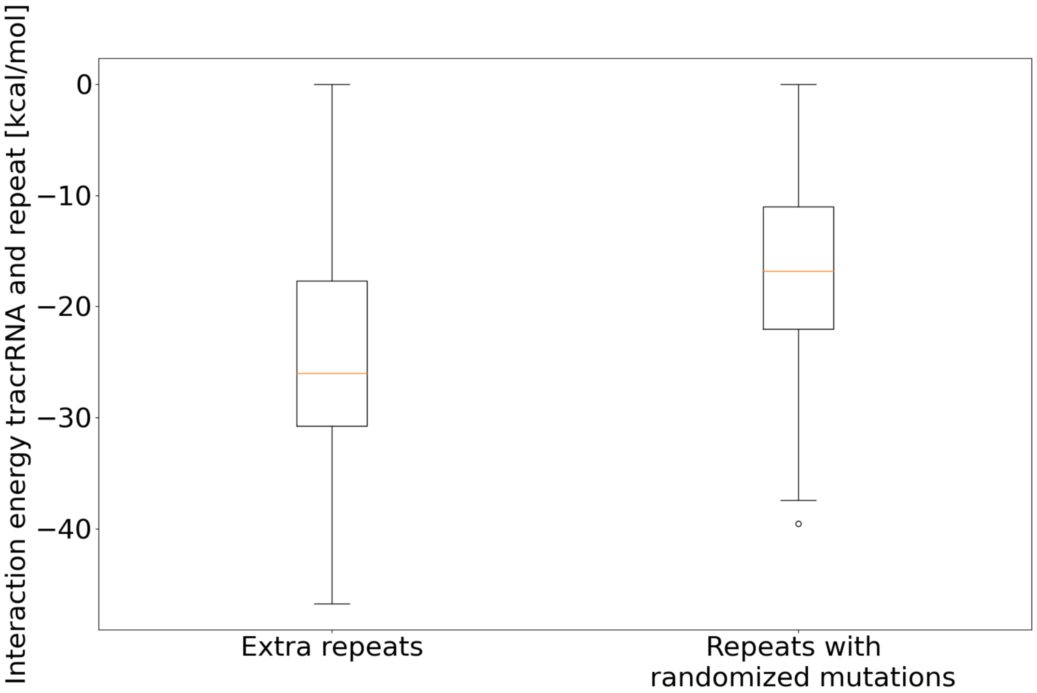

Figure S3: Interaction energy between tracrRNA and the extra (left) and repeats with randomized mutations (right). For each extra repeat, 20 repeats were randomly mutated with the same number of mutations as the extra repeat. 69 type II-C CRISPR-Cas systems were analyzed. Repeats with randomized mutations have a lower interaction energy than their respective extra repeats. This observation can be attributed to the observation that the majority of natural mutations occur at the 5' end (Fig. 2B) of the extra repeat, where the majority of bulges in the tracrRNA-repeat duplex also occur. Consequently, these mutations are more likely to be tolerated. Additionally, in certain instances, the initial nucleotides at the 5' end of the extra repeat do not contribute to the tracrRNA-repeat interaction[33–36].

Table S1: Type II-C repeats sorted based on the number of found mutations in the repeat.

mutations	0	1	<5	<10	<15	>15
Non-last repeat	6955	191	39	11	1	0
Last repeat	144	51	81	54	19	6

2
